# Estimating Economic Costs Associated with Treating Antimicrobial-Resistant Infections in Humans in Malawi

**DOI:** 10.3390/tropicalmed11100276

**Published:** 2026-09-30

**Authors:** Edward B. Masoambeta, Ganizani Nansongole, Luigia Scudeller, Tomislav Kostyanev, Finola Leonard, Fiammetta Bozzani, Eric Umar, Chisomo L. Msefula, Chantal M. Morel, Jobiba Chinkhumba

**Affiliations:** 1 Health Systems and Policy Department, Kamuzu University of Health Sciences, Blantyre 312200, Malawi; eumar@kuhes.ac.mw (E.U.); jchinkhumba@kuhes.ac.mw (J.C.); 2 Children Foundation Malawi, Monitoring and Evaluation Department, Baylor College of Medicine, Balaka 302100, Malawi; gnansongole@baylorfoundationmalawi.org; 3 Fondazione IRCCS Policlinico San Matteo di Pavia, Viale Camillo Golgi 19, 27100 Pavia, Italy; 4 Laboratory of Medical Microbiology, Vaccine and Infectious Disease Institute, University of Antwerp, Antwerp, Universiteitsplein 1, 2610 Wilrijk, Belgium; tomislav.simeonov.kostyanev@rsyd.uk; 5 School of Veterinary Medicine, University College Dublin, D04 V1W8 Dublin, Ireland; nola.leonard@ucd.ie; 6 Department of Global Health and Development, London School of Hygiene and Tropical Medicine, London WC1E 7HT, UK; fiammetta.bozzani@lshtm.ac.uk; 7 Pathology Department, Kamuzu University of Health Sciences, Blantyre 312200, Malawi; cmsefula@kuhes.ac.mw; 8 Institute for Public Health & Hygiene, University Hospital Bonn, 53127 Bonn, Germany; chantal.morel@ukbonn.de; 9 Liverpool School of Tropical Medicine, Pembroke Place, Liverpool L3 5QA, UK

**Keywords:** antimicrobial resistance, economic burden, economic costs, healthcare costs, instrumental variable regression, lost earnings, low- and middle-income countries

## Abstract

Background: Antimicrobial resistance (AMR) poses a significant threat to global health and economic development. In Malawi, the economic costs associated with the treatment of AMR infections remain largely unknown which may hinder resource allocation and policy prioritization. Method: The study used a cross-sectional design and was conducted between September 2023 and January 2025 at three different hospital settings. Data were collected from inpatients with laboratory-confirmed urinary tract infection (UTI) or blood stream infection (BSI). Additional follow-up data were collected from patients for four weeks post discharge. We analyzed patient level data first using descriptive statistics and employed an instrumental variable 2-stage least squares regression to estimate drivers of AMR treatments costs. Results: A total of 610 patients participated in the study. Of these, 45% had AMR-associated resistant infections. Overall, *E. coli* (43%) followed by *P. aeruginosa* (22%), *K. pneumoniae* (14%), and *S. aureus* (10%) were the most common pathogens. UTI was marginally more common (51%) compared to BSI (49%). BSI had a higher average cost than UTI for patients with both susceptible and resistant infections. On average, a patient spent MK283,815.10 (US$163.71) ranging from zero to MK13,500,000 (US$7786.90). Patients with resistant infections incurred, on average, MK161,785 (US$93.32) more than patients with susceptible infections, largely driven by prolonged hospitalization among other factors. Overall, the direct medical observed costs averaged MK37,165.29 (US$21.44) while direct non-medical observed costs averaged MK61,908.71 (US$35.71). Productivity losses for the patient and guardian(s) were a major observed cost averaging MK160,322.80 (US$92.49). The regression results further showed that having a bank account as a proxy of wealth, non-farm business, longer travel duration, BSI, and *K. pneumoniae* as the causative pathogens were significantly associated with increased cost burden among the patients. Conclusions: AMR-associated infections impose substantial economic burden on patients and households. Further strengthening of AMR surveillance and stewardship are needed as critical elements of Malawi’s National Action Plan. However, measures should also be taken to mitigate the economic consequences related to AMR, especially for patients who bear the brunt of these costs for lack of financial protection in Malawi’s health system.

## 1. Introduction

Antimicrobial resistance (AMR) has health and economic consequences for humans, animals and the general environment. While the health burden is well investigated with many published papers from both developed and developing countries [1,2,3], progress on the economic aspect remains limited even though there is growing recognition that global projected AMR costs are very high. For example, AMR costs are projected to be in the range of $300 billion to more than $1 trillion annually by 2050 worldwide [4].

The Centers for Disease Control and Prevention(CDC) estimates the costs of AMR at $55 billion every year in the United States: $20 billion for health-care and about $35 billion for loss of productivity [3]. Studies show that annual global GDP could decrease by approximately 1% and there would be a 5–7% loss in developing countries by 2050 [5,6]. If there is no change in the trends of AMR, the world will lose about $28 billion of its GDP in ten years of which $20 billion would be lost in the European Union and the Organization for Economic Cooperation and Development (OECD) countries. The direct impact of antimicrobial resistance on GDP is largely due to effects on labour through the loss of productivity caused by sickness and premature death [4]. The World Bank report demonstrates that global exports could decrease significantly by 2050 due to the effects of antimicrobial resistance in labour-intensive sectors [6]. 

Healthcare costs, as a direct monetary effect of AMR, are usually high and include expensive treatments that require intensive use of resources. Treating patients with resistant infections compared to other patients may require longer hospitalization stays, possible hospitalization in intensive care units (ICUs), treatment in isolation beds to prevent the spread of the infection, and use of more expensive treatment regimens [3]. Resistant pathogens may cause nosocomial outbreaks of infections leading to other indirect but costly outcomes such as the closure of an entire wing or unit of a hospital and the cancellation of elective surgeries, which can represent major losses for hospitals [7].

In addition to direct monetary effects, there are secondary effects that AMR generates as a burden on the health care system. These effects occur when the procedures that utilize antibiotics, which are essential to decrease the risk of any infection after surgery, cannot be successfully carried out due to high prevalence of AMR [2]. For instance, a comprehensive review on different multi-resistant infections that may occur after liver transplantation showed that multi-drug-resistant pathogens increase the likelihood of transplant failure and death [8]. However, data are limited on the exact costs of different secondary effects of AMR which limits our understanding of the scale of the problem [9].

AMR’s detrimental effects are not just limited to humans. According to the World Bank, AMR could have drastic impacts on livestock production in low- and middle-income countries [5]. Recent estimates of projected antimicrobial usage (AMU) show a 30% increase by 2040 [10] while indicating that if the persistent trends in AMR do not slow down, there will be an 11% loss in livestock production by 2050. Such a substantial loss in animal production would lead to a decline in income generation for farmers and the overall economic situation in affected countries. As in humans, the effect of AMR on animals is due to increased mortality and morbidity. The increase in resistance to antimicrobials renders treatments on animals ineffective and may increase the severity of infection [5]. Ultimately, this leads to decreased production and trade of livestock, resulting in elevated prices of protein due to the decrease in protein sources such as milk, egg, and meat. Shortage of protein could be a major concern, considering that the demand for animal proteins from meat, milk, and eggs among other products is on the rise worldwide [2,11,12]. Furthermore, detection of resistant organisms in food chains, can lead to major costs such as direct animal loss, supply chain disruption, market bans or restrictions, and farm closures. This effectively results in increasing labour costs and high prices of animal products that are in short supply [11,13].

The World Bank report shows that antimicrobial resistance would elevate the rate of poverty and impact low-income countries compared to the rest of the world [5]. Furthermore, due to AMR, the gap between developing countries and developed countries will become more pronounced; as a result, inequity will substantially increase [2]. Most of the people who are pushed into extreme poverty as a result of AMR will be from low-income countries. This highlights the fact that the underprivileged population of the world will eventually be affected the most because they are likely more dependent on labour income which will be reduced if there is a high prevalence of infectious diseases [2].

In Africa, resistant organisms have acquired further resistance against a wide array of antimicrobials over the years, substantially escalating costs of treatments, overall costs and resources. For instance, AMR bacterial bloodstream infections are among the common causes of morbidity and mortality in sub-Saharan settings, yet few facilities are able to maintain long-term surveillance of affected patients to assess their full burden and impact [14]. From 1998 to 2016, over 50% of bacteria isolated from blood samples of patients at Queen Elizabeth Central Hospital (QECH) in Blantyre Malawi were resistant to the first line antibiotics recommended by national guidelines suggesting that treatment would fail [14]. 

Most AMR related deaths occur in low- and middle-income countries (LMICs) [15,16], including Malawi. In LMICs, AMR-associated BSIs and gastrointestinal tract-related bacteriologically confirmed infections caused by drug-resistant organisms have increased rapidly by a 58% rise in mortality, with AMR-associated BSIs alone causing direct medical expenses of up to USD 12,000 per patient [17]. Similarly, a large proportion of deaths in Malawi remain attributable to infections, a problem magnified by emergent antibiotic-resistance [18].

In Malawi, the rapid increase in the prevalence of *Enterobacterales* producing extended Spectrum Beta-Lactamase (ESBL) among common Gram-negative pathogens, and the emergence of Methicillin-resistant *Staphylococcus aureus* (MRSA) as causes of BSI, highlight the growing challenge of infections that are effectively impossible to treat in such resource-limited settings [19]. 

To support interventions aimed at combating the rise in resistant pathogens, Malawi developed and launched The Malawi AMR National Action Plan (NAP) running from 2017 to 2022. The Malawi NAP has five thematic areas including education and awareness, surveillance and research, infection prevention and control, optimal use of drugs, and investment and sustainability [15,20]. There remains an urgent need for context specific empirical evidence to inform and justify investment across all five thematic areas. Implementation of the NAP was slow and almost entirely dependent on donor-initiated projects, resulting in little progress in some key themes of the NAP [20]. At present, the economic costs associated with AMR are unknown as benchmark figures at the national level are not known. Also, there is a need for the use of robust economic costing methods to generate evidence at national level since many studies in Malawi have looked at either clinical or economic aspect of AMR separately or at a single site (hospital), using analytical methods (e.g., thematic, and descriptive) or quantitative methods that do not consider confounding or impact evaluation of the full economic burden of AMR which may lead to biased results and hence inappropriate policy decisions. Consequently, this study aims to contribute to the estimation of the economic burden of AMR in humans within a One Health framework to complement efforts through the known cost burden.

**Figure 1:**
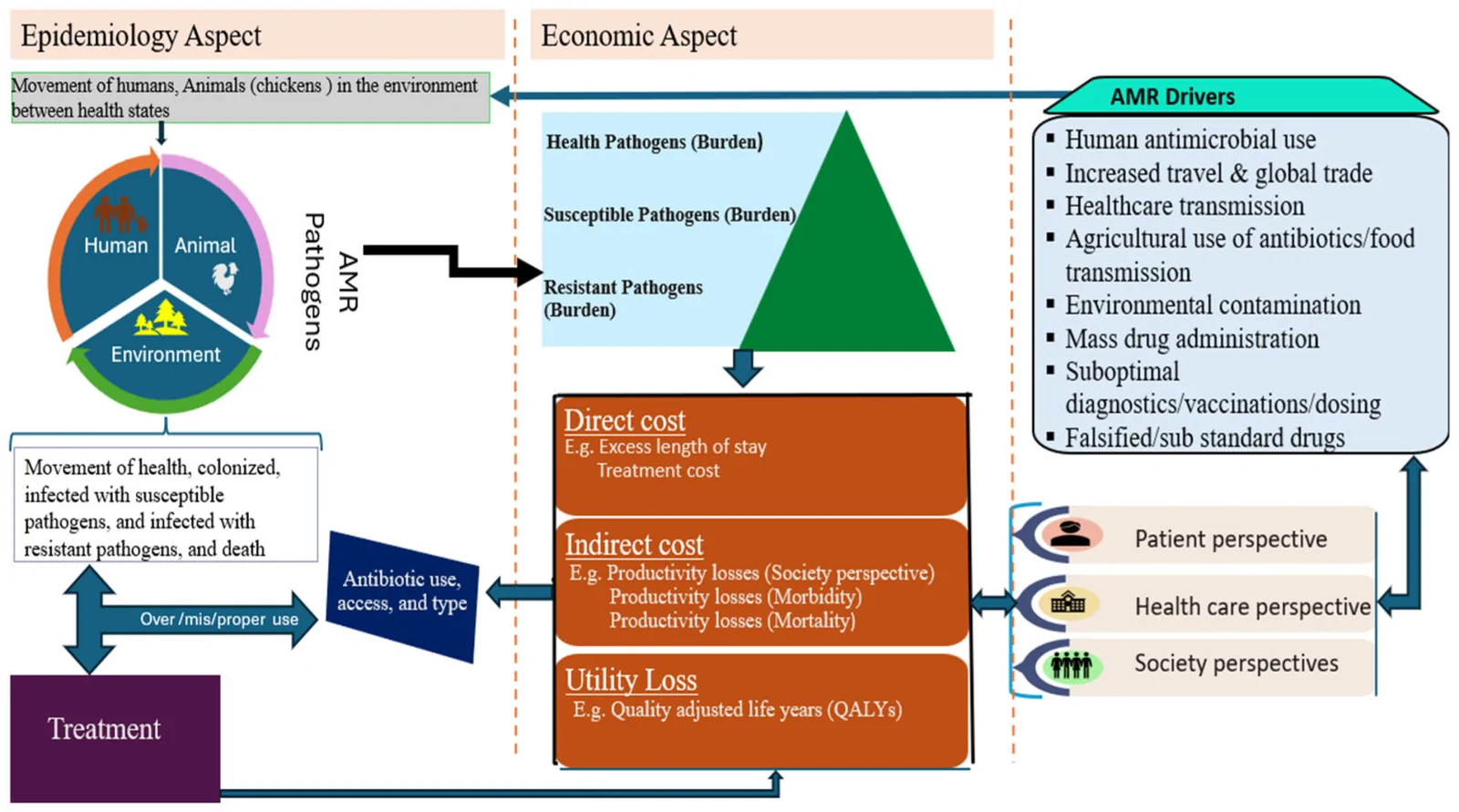
AMR epidemiology and economic costs framework.

The interrelationships between AMR in One Health settings, epidemiology, and economic costs can be summarized using the framework shown in Figure 1.

Due to the AMR burden as a result of AMR drivers in any of the sectors, certain costs will arise. At the individual level, there will be increased morbidity and mortality, associated lost earnings, and direct non-medical and patient care costs due to longer admissions. At the healthcare level, there are costs of surveillance and containment programs, longer hospital admissions, reduced capacity due to isolation requirements, and additional diagnostics due to resistant infections among others. The societal levels’ costs include the productivity losses, reduced incomes from trade and tourism due to poor quality meat from animals and reduced production as a result of morbidity and mortality in humans.

In this antimicrobial resistance (AMR) model, income serves as a proxy for an individual’s or producer’s ability to pay for healthcare and veterinary services. Higher income directly lowers the financial barrier to accessing antibiotics, enabling greater utilization across multiple sectors including human medicine and livestock treatment. While this access is necessary for treating infections, it simultaneously increases the overall volume of antibiotic consumption. Critically, as affordability rises, so does susceptibility to misuse and overuse, since financial constraints no longer act as a check on prescribing or purchasing practices. This elevated and often inappropriate use intensifies selective pressure on pathogens, thereby accelerating the development and spread of resistance, whether in colonized or infected humans, treated animals, or contaminated environmental reservoirs requiring intervention [21,22].

Also, many other healthcare interventions such as vaccinations, diagnostics, and non-antibiotic treatments, affect antimicrobial consumption, and consequently increase or decrease the risks of AMR [23,24].

The framework used in this publication is inspired by the Agriculture Human Health Micro-Economic model (AHHME) which uses Markov state transition models to model the movement of humans and food animals between health states [25]. This model shows the rather complex set of potential direct and indirect AMR pathogen transmission paths among humans, animals, and the environment in different states fueled by selected forces (the drivers) and the implications on costs at different impact levels (individual, healthcare or provider, and society) over time.

Humans, animals and the environment interact continuously through multiple activities that facilitate the transmission of AMR pathogens. These interactions may occur directly between humans and animals or indirectly through environmental pathways. Figure 1 shows that the movement of pathogens is economically less burdensome if transferred between healthy animals, humans, or the environment compared to the movement of resistant organisms in infected or diseased individuals. Holmes et al., (2016) and Wall (2016) showed that an increase in activities leading to increase in any of the AMR drivers, has an impact on the cost burden at the individual or health-care, and societal levels [26,27].

The framework allows for tracking of costs, drivers, and epidemiological burden paths in One Health, specifically the human component in this publication. In low- and middle-income countries, the framework must account for higher mortality rates and less advanced treatment facilities, often leading to lower direct costs but higher indirect losses. Evidence from the literature showed that AMU restriction in animals was associated with decreases in ARB in humans and the environment, which supports the need to tackle AMR from a One Health perspective [16,17,28].

With the aim of informing the estimation of the overall AMR cost burden, this study focuses on the costs deriving from associated resistant infections in humans across secondary and tertiary health care levels. The specific objectives were (1) to estimate the magnitude of observed economic costs associated with AMR infections, (2) to determine the causal effect of resistance and the drivers of observed economic costs attributable to AMR-associated infections, and (3) to estimate the incremental cost of resistant versus susceptible AMR-associated infections via total observed treatment costs.

## 2. Materials and Methods

A cross-sectional study was conducted to collect data from patients with laboratory-confirmed AMR-related bloodstream infections (BSI) or urinary tract infections (UTI). The study sites were three public hospitals in Malawi purposively selected for their established AMR surveillance systems and their geographical locations, representing all three regions of Malawi: Zomba Central Hospital and Kamuzu Central Hospital (tertiary), and Rumphi District Hospital (secondary), for the Southern, Central, and Northern regions, respectively. Written ethical approvals were obtained from the Kamuzu University of Health Sciences and all the three hospitals after the presentation of the research protocol to the research ethics teams/committees.

The study population comprised patients seeking care for recent UTI or BSI known to be resistant based on laboratory testing and confirmation, following two of the clinical breakpoints from the European Committee on Antimicrobial Susceptibility Testing (EUCAST): susceptible (S) or resistant (R). Isolates recovered from the blood or urine samples were tested against the WHO recommended Accessible, Watch, and Reserve (AWaRe) antibiotics using disk diffusion method. The antibiotics used included Amikacin, Amoxycillin, Ampicillin, Doxycycline, Tetracycline, Cefepime, Ceftazidime, Ceftriaxone, Ciprofloxacin, Levofloxacin, Clindamycin, Lincomycin, Chloramphenicol, Azithromycin, Sulphathiazole, Trimethoprim, Vancomycin, Gentamicin, and Meropenem. The concentrations of antibiotics in the disks were as shown in Appendix C. Participants were allocated to either the S or R group based on whether the patient was treated with an antibiotic that subsequent laboratory testing showed the organism was resistant to. The susceptible group comprised the patients whose laboratory confirmed results showed high likelihood of treatment success using all available AWaRe antibiotics. The resistant-infection group had 245 patients while the susceptible group had 365 patients. The health information management system (HIMS) records for 2021 and 2022 indicated approximately 667 AMR infection cases annually at both Zomba Central Hospital and Kamuzu Central Hospital, while about 152 cases were recorded at Rumphi District Hospital. These are used as population for Zomba (N = 667), Kamuzu (N = 667), and Rumphi (N = 152). We then used The Yamane formula for finite populations specified as follows;

(1) $$n=N/(1+N\left(e^{2}\right)\frac{{NZ}^{2}\left(p\left(1-p\right)\right)}{\left(N-1\right)e^{2}+Z^{2}\left(p\left(1-p\right)\right)})$$

where:

n = Required sample size

N = Known population size

Z = Standardized score for 95% confidence interval

e = Margin of error (precision level), 0.05.

The formula gives a calculated sample size of 500 for Zomba and Kamuzu central hospital, and 110 for Rumphi, giving a total sample size of 610.

Data collection occurred in two phases. First, patients were identified via hospital laboratory registers. Following identification and consent, baseline data were collected using a structured questionnaire to ensure high reliability and validity through exactly the same pre-defined choices (such as Yes/No or Likert scales) and a low risk of interviewers’ own interpretation of responses. The questionnaire was programmed in the KOBO Collect platform (ODK Collect v2024.3.4 downloaded from https://play.google.com/store/apps/details?id=org.koboc.collect.android, accessed on 2 August 2026) and was piloted to check its functionality. Enumerators, trained nurses, collected socio-demographic and initial cost data. One month after discharge, a follow-up interview was conducted to capture complete cost data, including any extra treatment expenses, out-of-pocket expenses, transport costs, lost earnings, and death-related costs in-cases of death of the patient.

Micro-costing at the patient level captured direct medical costs, direct non-medical costs (transport, food), and indirect costs (productivity losses).

This study employed an instrumental variable (IV) 2-stage least squares (2SLS) regression estimation technique for data analysis inspired by the econometrics work of Heckman and Goldberger (1972) [29] with the following structural or second stage model specification:

(2) $$lnY_{i}=\beta_{0}+\beta_{1}X_{1i}+\ldots +\beta_{k}X_{ik}+\alpha_{1}{\hat{y}}_{1i}+W_{i}\gamma +\varepsilon_{i}$$

where;

$ln\;Y_{i}$ is the dependent variable measured as natural log (ln) of total cost of AMR-associated infection in Malawi Kwacha (MK) incurred by individual i. These costs were derived by accounting for all costs incurred by a patient, which included all hospital costs such as direct medical costs, productivity losses using the human capital approach via functional time of the patient or guardian, transportation costs, death-related costs associated with AMR infections, and all other indirect costs.

$X_{1i}$ to $X_{ik}$ is a set of exogenous factors that affect AMR total costs which included age, sex, health insurance, education, occupation, travel duration, wealth, type of infection, and causative pathogens,

${\hat{y}}_{1i}$ is a proxy, as the predicted value of the endogenous variables, resistance, illness duration, and the interaction term of the two in our case,

$W_{i}$ is a vector of other of control variables such as squared and interaction terms of the exogenous factors, and

*β*_0_, $\beta_{i}$, $\alpha_{1},$ γ, and $\varepsilon_{i}$ are for a constant, slope coefficients of exogenous factors, parameter to measure the causal effect of ${\hat{y}}_{1i}$ on $ln\;Y_{i}$, the (predicted) value of endogenous variables, parameter to measure influence of control variables on $ln\;Y_{i}$, and-error term of the regression model respectively.

The first stage regression equation is modelled with our endogenous variable $y_{1i}$, which is the resistant-infection variable in our case, as a dependent variable of all other exogenous variables, including the IV(s), excluded in the second stage. The following is the specification:

(3) $$y_{1i}=\varphi_{0}+\varphi_{1}X_{1i}+\ldots +\varphi_{k}X_{ki}+\pi_{i}Z_{i}+W_{i}\vartheta +v_{i}$$

where;

$y_{1i}$ is the endogenous variable for resistant infections,

$X_{1i},\ldots ,\;X_{ki},\;and\;W_{i}$ are the same regressors of the second stage equation,

$Z_{i}$ represent instrumental variable(s) excluded in second stage, length of hospital stay and interaction term of resistance and length of hospital stay in our case,

$\varphi_{0}$, $\varphi_{1}$, $\pi_{i}$, are estimated parameters in the equation of which $\pi_{i}$ must be statistically significant if the instrument is valid, and, $v_{i}$ is the error term of the regression equation.

The IV 2sls further assume that the expectation of the error term is zero and is uncorrelated to the instrument(s), that is, $E(v_{i}$) = 0, and $Cov\left(V_{i},\;Z_{i}\right)=0$.

The study used STATA 16.0 (from StataCorp. 2019, College station.TX downloaded from https://www.stata.com) to analyze the data and to carry out regression diagnostic tests. The IV 2SLS estimation technique was used to analyze the economic cost burden of AMR using command *ivregress 2sls*. We chose IV regressions for a number of reasons, such as the advantage of incorporating tests of assumptions about the exogeneity of treatment effects in the data while allowing for experimental design effects. Also, this estimation technique was helpful to deal with the endogeneity problem when present in the model. For example, there is an estimation problem when AMR may cause prolonged hospital stay/duration of suffering, with catastrophic expenditures increasing the costs associated with the infection and at the same time, the type of antimicrobials in use may affect the duration of the infection which later exacerbates the costs associated with AMR, be it direct (like clinical tests) or indirect (lost earnings) [2,30,31]. Again, due to standard treatment guidelines, the prescription and administration of antibiotics to treat AMR infections after laboratory tests may show statistical properties of non-random behaviour in the data. This, coupled with unobserved patients’ behaviour such as adherence of prescribed dosage of taking antibiotics, or undisclosed use of earlier antibiotics bought from medicine stores, or indeed where laboratory tests are not done due to lack of resources and resort to use presumptive treatment guidelines may all pose as unobserved data in studies like these further creating problems of estimation such as endogeneity and confounding [32,33,34].

Interestingly, IV 2SLS methods can yield unbiased treatment effect estimates in the presence of unobserved or unmeasured confounding provided the sample size is sufficiently large according to John et al., who assessed causal treatment effect estimation when using large observational datasets [35]. This calls for a proper estimation technique such as IV 2SLS.

The regression estimation was subjected to a number of tests to check for statistical significance, as well as to assess the assumptions of the model and/or data problems. The researcher tested the endogeneity of the variable instrument variable validity using the probability value of both the F-statistic and the partial R-squared. Additionally, a test on over identification of restrictions and model specification was done using Sargan test. Furthermore, heteroscedasticity test was also carried out to check for the variability in the variance of the error term.

All monetary values are presented in Malawi Kwacha (MWK) and converted to United States Dollars (USD) where applicable using the official mid-market exchange rate of 1 USD to 1733.68 MWK for the last quarter of 2025 as quoted by the Central bank, the Reserve Bank of Malawi.

## 3. Results

### 3.1. Descriptive Statistics

Table 1 shows the descriptive statistics of the variables used in the study—with a sample size of 610 patients—except for the *death cost*, whose cost data are associated with 25 deceased patients.

Descriptive statistics results show that the patients average age was 42 years with a 95% confidence interval (CI) of 40.01 to 43.20. Most of the study respondents were females (55%) with the majority living in urban locations (65%, [0.61; 0.69 95% CI]). Only 6% of the patients were on medical schemes with 95% CI of 0.04 to 0.08.

In terms of the level of education of the patients in the study, about 43% [0.39, 0.47 95% CI] were educated up to primary level (or not beyond grade 8) with 29% [0.26, 0.33 95% CI] educated up to secondary level. The minority (about 12% with 0.10, 0.15 95% CI) had tertiary education.

In terms of occupation, 36% [0.32, 0.40 95% CI] were not employed, 29% [0.26, 0.33 95% CI] were farmers, while the minority of 14% [0.11, 0.17 95% CI] were civil servants among other occupations. Bank account ownership, an index of wealth in the study, indicated that 48% [0.44, 0.52 95% CI] of participants owned a bank account. The patients used motorbike transport (33%, 0.30, 0.37 95% CI) more than any other mode.

#### 3.1.1. Prevalence of BSI and UTI 

The study focused on BSI and UTI with results showing that 49% [0.45, 0.53 95% CI] of the infections were BSI. The duration of AMR infections was 21 days [18.76, 22.15 at 95% CI] for most patients on average with extreme and chronic infections lasting 3 to 8 months. The most common identified pathogen was *E. coli* (43%, 0.39; 0.47 95% CI) followed by *K. pneumoniae* (14%, 0.11; 0.17 95% CI), and *S. aureus* (10%, 0.07, 0.12 95% CI) overall. Pathogens associated with UTI alone were *E. coli* (52.87%, n = 138), *P. aeruginosa* (50.36%, n = 69), *S*. *aureus* (58.62%, n = 34), *K. pneumoniae* (47.62%, n = 40), and the rest (48.31%, n = 31). There was no difference in the prevalence of pathogens in UTI versus BSI (chi-square value of 3.38, *p* < 0.497) shown in Table 2 below as column percentages.

For BSI, 52.38% (n = 44) was caused by *K. pneumoniae*, 49.64% (n = 68) by *P. aeruginosa*, 47.13% (n = 123) by *E*. *coli*, 41.38% (n = 24) by *S*. *aureus*, and 51.69% (n = 39) by other pathogens.

**Figure 2:**
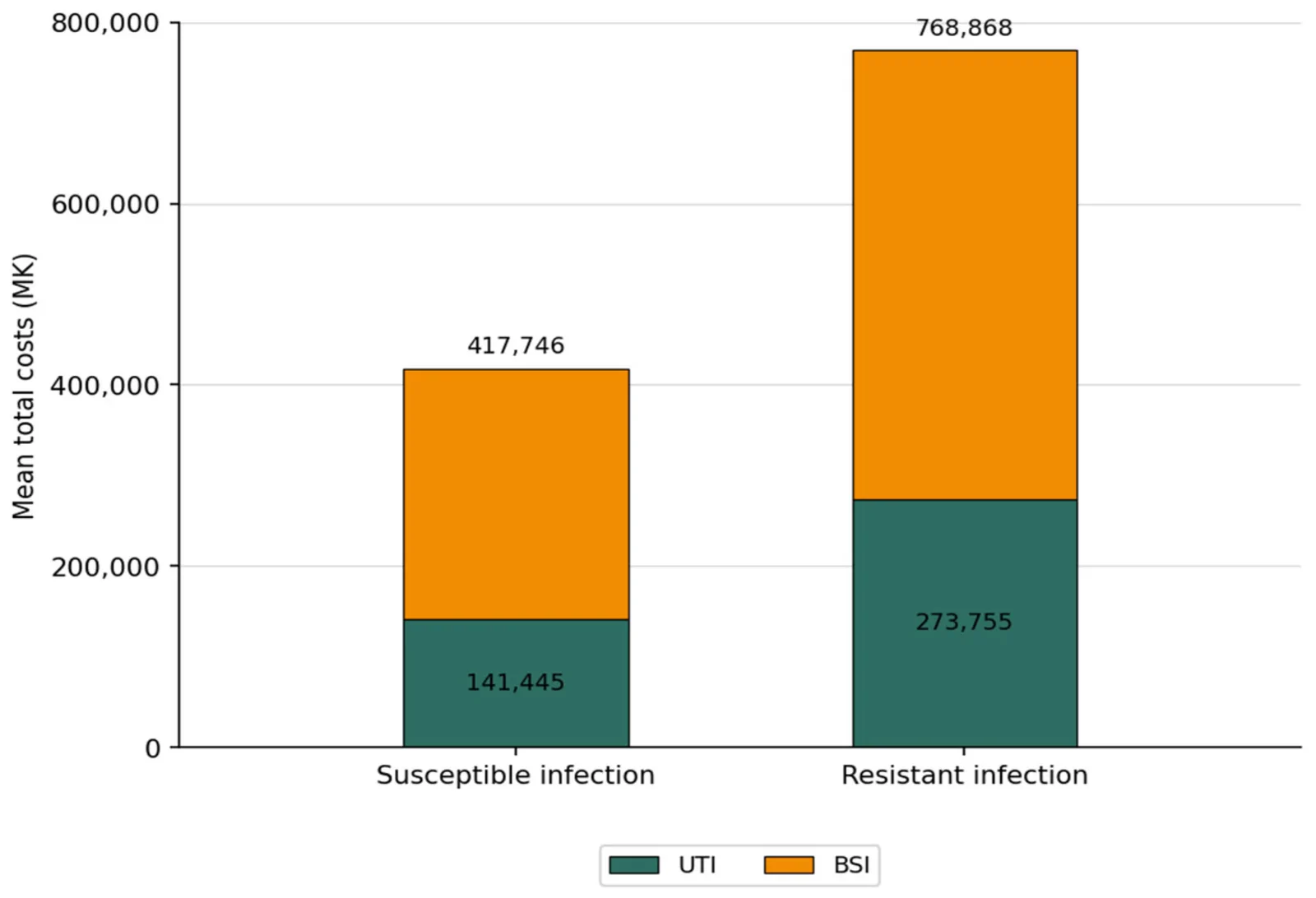
This figure compares the observed mean total costs between patients with pathogens resistant or susceptible to the antibiotics used for treatment of UTI or BSI.

Pathogen prevalence shows that organisms resistant to the antibiotic used for treatment were isolated from 45% [0.41, 0.49 95% CI] of the total infections and the organisms isolated from the remainder of infections were classified as sensitive to the antibiotics used for treatment. The majority of the resistant infections were UTIs at 66% while 53% of BSIs were susceptible to the antibiotics employed (Pearson chi (1) = 4.38, *p* < 0.036). In relation to total costs, the mean observed costs among patients with a resistant infection was MK161,785 (US$93.32) higher than among patients with a susceptible infection, as shown in Figure 2 below.

Specifically, the mean cost was MK132,310 higher for patients with a UTI caused by a pathogen resistant to the antibiotic used for treatment compared to a susceptible UTI, and MK351,122 higher in the case of BSI caused by an organism resistant to the therapeutic agent used versus a susceptible organism.

The majority of patients with *P. aeruginosa* infections were treated with antibiotics to which the organism was susceptible (79.56%) followed by other or mixed infections (64.73%), 53% *E. coli*, and 46.55% *S. aureus.* Table 3 below shows more details of the causative pathogens by infection category, presented as row percentages.

On the other hand, resistant infections were associated with 57.14% of *K. pneumoniae,* followed by *S. aureus* (53.45%), *E*. *coli* (46.74%), and 20.44% of *P. aeruginosa*. These results were statistically significant (*p* < 0.000).

#### 3.1.2. BSI and UTI Treatment Costs

At the patient level, cost descriptive statistics show that patients spent an average of MK14,211.43 (US$8.20) and a maximum of MK253,000.00 (US$145.93) on transport and the majority (62%) spent less than one hour when visiting the health care facilities. Lost wages averaged MK112,625.60 (US$64.96) with a maximum expenditure of MK1,280,000.00 (US$738.31). The observed average cost of treatment was MK37,165.29 (US$21.44) and a maximum of MK786,000.00 (US$453.37). Additional costs on items such as food and airtime averaged MK47,697.27 (US$27.51) and a maximum of MK2,000,000.00 (US$1153.62). The costs related to death of the 25 patients during the follow-up period of one-month averaged MK253, 040.00 (US$145.96), with a minimum expenditure of MK64,000.00 (US$36.92) and a maximum of MK700,000.00 (US$403.77). Death associated costs included mainly the cost of coffin, food, and transport. Thus, in total, a patient spent MK283,815.10 (US$163.71) on average as a result of 20.5 days of AMR-associated infection. The observed total cost reached as high as MK13,500,000 (US$7786.90) while others incurred no costs.

#### 3.1.3. Direct Medical, Direct Non-Medical, and Productivity Losses

The costs were further classified into direct medical, direct non-medical, and productivity losses. In this section, we first differentiate these costs based on the timing of collection—that is, before follow-ups and during the follow-ups—and lastly, we look at the combined costs. Follow-up was done one month after discharge of a particular patient. 

**Figure 3:**
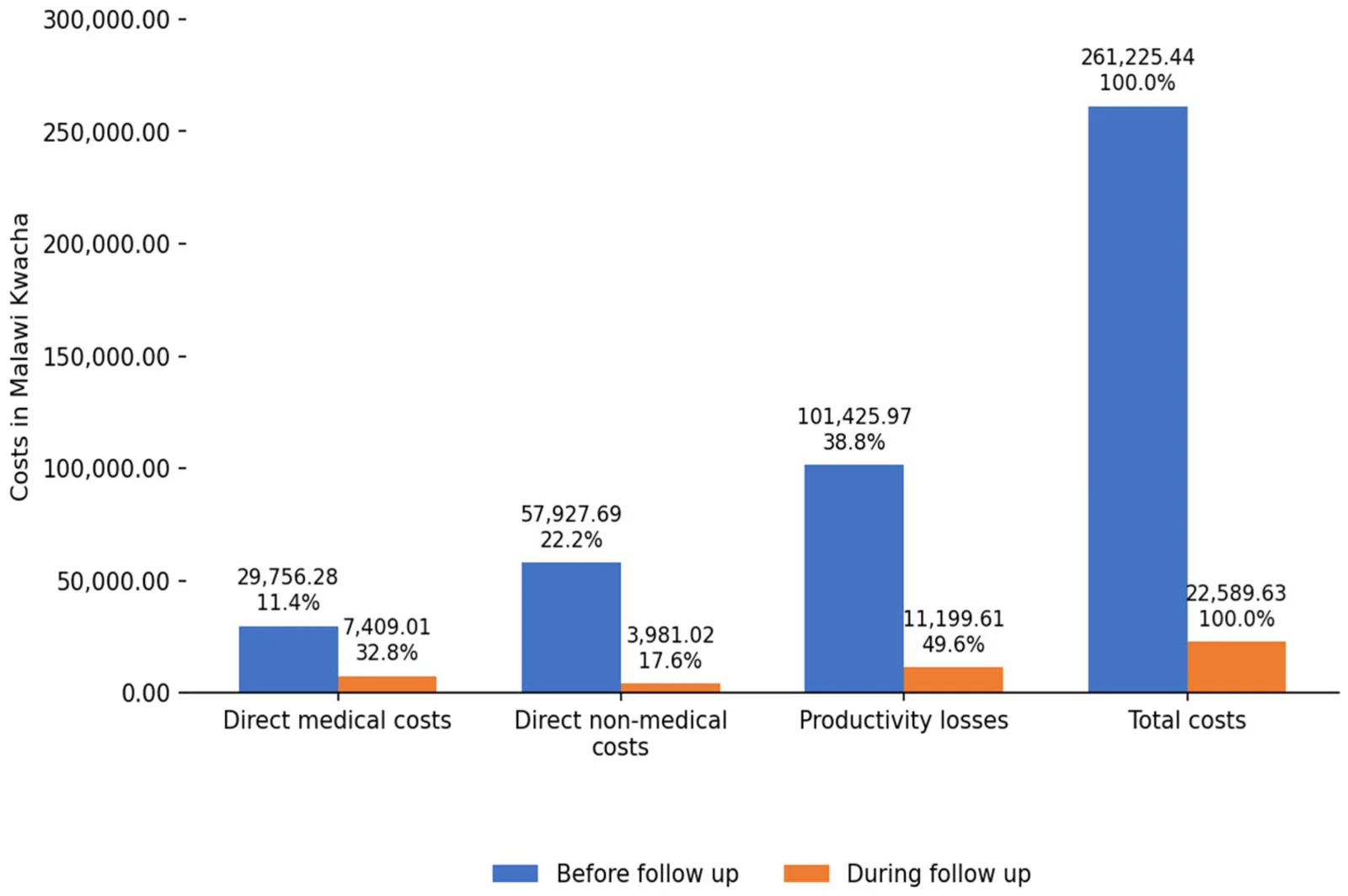
This figure shows the details of observed mean and percentage costs before and during follow-ups.

The mean costs before follow-ups are the highest across direct medical, direct non-medical, and productivity losses compared to during follow-ups, with observed productivity losses at MK101,425.97 (US$58.50) and direct medical costs at MK29,756.28 (US$17.17) as the highest and lowest expenditures, respectively. These costs are shown in Figure 3 below.

The costs for follow-ups show the highest observed mean productivity losses (MK11,199.61 or US$6.46) and the lowest observed mean direct non-medical costs (MK3981.02 or US$2.30).

In Table 4, the overall direct medical costs averaged MK37,165.29 (US$21.44), while direct non-medical costs such as transport and food, averaged MK61,908.71 (US$35.71).

Productivity losses for the patient and guardian(s) were a major cost, averaging MK160,322.80 (US$92.49) while costs associated with AMR crude mortality were MK6326,000 (US$3648.89) in this study.

**Figure 4:**
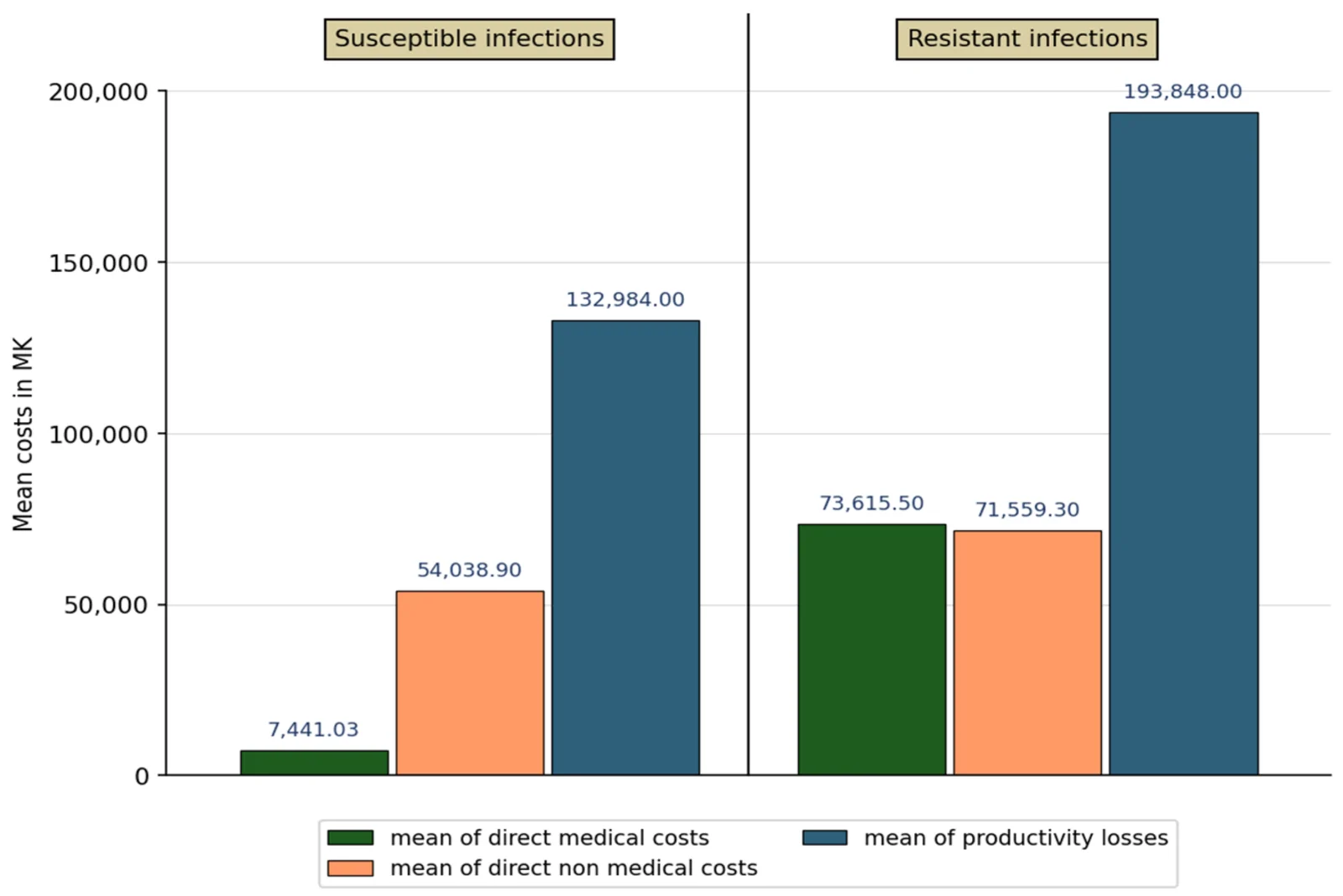
This figure shows a comparison of cost types associated with types of resistance of infection.

When compared across susceptible and resistant infection groups, all cost categories are much higher for the latter than for the former. Specifically, the mean direct medical costs, direct non-medical costs, and productivity losses for the resistant infection group were 9.9, 1.3, and 1.5 times higher relative to susceptible infection group respectively as shown in Figure 4.

Clearly, it is more burdensome to have a resistant infection than a susceptible one as all economic costs are much higher for the former group than the latter. Across the cost categories, productivity losses are the highest costs that patients incurred followed by direct non-medical and then direct medical costs. The direct medical costs are relatively much higher for a resistant compared to a susceptible infection which highlights a higher burden and hence additional support in terms of resources.

The direct medical costs could have been much higher if the study had included all services, drugs, and private hospitals as some services at the government hospital are free or subsidized. Nonetheless, the researcher converted most of the standard prices used from the Malawi Medical Stores website which is a government agency responsible for the purchase and supply of government hospital accessories, including medicines (http://www.cmst.mw/catalogue/cats/bb_class.php, accessed on 3 November 2025). Also, some patients could not recall treatment related exacerbation by missing patient registers and health passport books sometimes due to prolonged periods of suffering.

### 3.2. Instrumental Variable 2-Stage Least Squares Regression

We employed an IV 2SLS regression technique using patient level data to solve the problem of endogeneity which was confirmed in the model. We found that the resistance-status variable of the AMR-associated infections, a regressor, was endogenous which is instrumented by the length of hospital stay and the interaction term between resistance and length of hospital stay variables. Using the Durbin chi-square (*p* < 0.0387) and Wu-Hausman (*p* < 0.0423) tests (Appendix A), we reject the null hypothesis that the resistance variable is exogenous and confirm that it is endogenous which justifies the use of IV regression.

This was expected because studies like Otieku et al., 2023, and Dadgostar et al., 2019, found that as resistance levels rise, the original antibiotics become less effective, which in turn demand the use of newer, often more expensive or broad-spectrum drugs to treat infections [2,36,37]. This ongoing use of different antimicrobial agents continues the cycle, selecting for even more complex multidrug-resistant strains. This implies that the length of hospital stay and interaction with resistance itself is an important instrument in the event of unobserved antibiotics in use, just like in this study where patients were unable to clearly recall details themselves and the health passport or hospital registers only showed limited information for various reasons, including loss and inability to trace patients’ information especially on referrals.

Some patients also indicated that they stopped using antibiotics because they felt better, or bought themselves antibiotics from drug stores without a prescription from a doctor. Again, the use of antibiotics in terms of type may not have varied much among patients due to standard treatment guidelines given laboratory outcomes and availability of required antibiotics. When the availability of various types of antibiotics is constrained, as in resource-limited settings like Malawi, the problem of endogeneity and confounding is more likely, as physicians prescribe or administer the same alternative antibiotics for different types of infections, which may not be effective enough to treat the infection at hand, leading to longer durations of treatment and hence increased costs. These problems are among the drivers of AMR which Shrestha et al. (2018) summarized as either sub-optimal dosing or mass administration of antibiotics [24].

Sub-optimal management of antibiotics in LMICs, including the overuse, is promoted by misconceptions, social-cultural issues, diagnostic uncertainty, and clinical competency as well as commercial, patient, and time pressures [38]. Therefore, all these factors should ideally be considered when estimating costs, but—although impactful—they are difficult to observe and model.

Furthermore, we ran the first stage regression to check the desirability of running IV regression with resistant infection variables being endogenous along with other exogenous factors. The adjusted R squared (0.66) and F-statistic (479) of first stage regression indicates a well fit model with 66 of the variations in log of total costs being explained by the exogenous and endogenous variables which are jointly statistically significant as the F-statistic (479) is way above 10 (Appendix A). The Wald test applied to the regression result at 5% with a minimum eigenvalue statistic of 478.78 also confirms that the instruments used are valid and strong.

The instruments were also subjected to Sargan and Basmann chi-square tests for overidentifying restrictions in the model which produced *p*-values of 0.2688 and 0.2723 respectively (Appendix B). The results show that the instruments were overidentified, as required for better results.

Lastly, to ensure that there was no heteroskedasticity in the variance of the regression error term, a robust IV SLS was run.

The dependent variable, total costs, was transformed via natural log (ln) to improve skewness (or normalize the distribution) of the cost data as some patients reported very high costs while others reported no costs. Thus, the data became approximately normally distributed through the natural log transformation, relative to the small values of other variables, especially the categorical ones.

The regression results in Table 5 (which actual Stata output is a supplementary material and can be downloaded at: https://www.mdpi.com/article/10.3390/tropicalmed11100276/s1, Table S1: Stata IV 2sls regression) show that a patient with a resistant infection (*p* < 0.000) incurred about 82% more costs than a patient with a susceptible infection over the mean 20.5-day period of AMR-associated infections. This implies that it is more costly to treat an antimicrobial infection when it is caused by a resistant bacterium than a susceptible one.

The results also indicate that having a bank account (*p* < 0.027)—a proxy of wealth—resulted in a patient’s ability to spend about 33% more money than one without a bank account, all else being equal.

The patient’s occupation, a categorical variable with a base category of ‘an unemployed patient’ in the regression estimation showed that a patient engaged in (non-farming) business (*p* < 0.002) incurred 66% more costs towards AMR infection treatment than one without a formal job. There was no statistically significant difference in spending patterns between a patient doing farming (*p* < 0.542) and the unemployed during the period of AMR illness. However, a patient in civil service would likely incur less costs than the unemployed but this result has a statistically weak support of evidence at the 5% standard level of significance (*p* < 0.092).

The travel duration is an ordered categorical variable measured in the range of hours of time it took to reach a particular health facility with a base category of ‘less than one hour.’ The results therefore imply that a patient who spent one to two hours travelling to reach a health facility (*p* < 0.000), incurred 75% more overall costs than a patient who spent less than one hour to reach the same healthcare facility, on average, *ceteris paribus*. Again, for a patient requiring more than 2 h to reach a particular healthcare facility (*p* < 0.000), the costs incurred increased by 136% compared to a patient who required less than one hour to visit the same facility, on average. Similarly, for a patient spending about 5 h to travel to a given healthcare facility (*p* < 0.015), the rise in costs was 129% more than a patient spending less than one hour, holding all else equal.

Additionally, regarding type of infection (as a dummy variable), the regression results indicate an increase in total costs incurred by a patient with BSI compared to a patient with UTI by a magnitude of 91% (*p* < 0.000).

Lastly, the causative pathogen, a categorical variable, is compared with patient infections caused by *E. coli.* The regression estimated outcome shows that a patient’s infection caused by *K. pneumoniae* (*p* < 0.001) resulted in 69.65% more total cost expenditure compared with a patient whose infection was caused by *E. coli*, on average, holding other factors constant. Also, a patient’s AMR infection caused by other pathogens (*p* < 0.001) other than *E. coli*, *S. aureus*, and *K. pneumoniae* or a multiple of these pathogens increased total costs by about 75% more than a patient’s total cost associated with AMR infection caused by *E. coli* alone.

## 4. Discussion

As anticipated, the resistance variable status of AMR-associated infections in the model was found to be endogenous, in which case standard linear regression would give biased estimates. Regression techniques, specifically, which handles endogeneity and confounding, for instance IV 2SLS employed in this study were found to be among robust methodologies during the systematic review of economic methodologies for AMR. Employing the IV 2SLS in this study has allowed for checking expected econometric data problems, endogeneity in our case, and remedies for reliable results. This is in agreement with studies by Iskander et al. (2021), and Ma et al. (2021) [39,40] which singled out potential endogeneity and confounding in AMR infection cost estimation. The study successfully addresses a key methodological challenge. The confirmed endogeneity of the resistance variable (via Durbin and Wu-Hausman tests) likely stems from the complex, bidirectional relationship between treatment choices, infection duration, and costs, exacerbated in settings with inconsistent antibiotic access and use [24]. Our use of infection duration and its interaction with resistance as instruments is statistically valid and clinically plausible, as prolonged illness directly escalates costs which may be a consequence of limited access and type of antibiotics and hence ineffective treatment of resistant infections.

The demographic data indicate a predominantly female (55%) and urban population, with a significant proportion of patients lacking health insurance (94%). This lack of coverage is concerning, as it exacerbates the financial burden on families and limits access to necessary medical care. Additionally, a patient’s occupation and wealth affect overall expenditure on resistant infections such that wealthier individuals and those in non-farm businesses are likely to spend more on treatment of resistant infections. These findings mirror very well the findings by Miller-Petrie that there is greater likelihood of wealthier individuals to access and choose among drugs, including new, last-resort treatments that are expensive and therefore unaffordable for lower income individuals [41]. The study further indicated contextual limitations for individuals in LMICs who are likely to face more limited access to quality drugs compared to high-income individuals [41]. This suggests that employment status directly influences the economic burden of health-related decisions and outcomes including AMR. As many patients cannot afford treatment due to lost income, targeted employment programs and social support systems may be necessary to alleviate this burden.

Among the findings of this study, a patient’s length of hospital stay averaged 20.5 days. This period is longer than a mean of 7.4 days that Poudel et., al found from a meta-analysis of 25 reviewed papers on economic burden of antibiotic resistance [42]. Other studies by Otieku et al. in Ghana and Oterino-Moreira in Spain, found AMR length of hospital stay to range from 12.3 to 16.6 days and 8 to 14 days respectively on average with resistant-infection cohort taking longer than patients with susceptible infections [36,43]. The case for Malawi has a longer average length of stay due to AMR compared with similar studies covered in this study. This aligns with global evidence as the magnitude is particularly contextual. For instance, a systematic review by Dadgostar (2019) noted that AMR infections consistently lead to longer hospital stays and higher costs, but studies from low-income countries often report more extreme relative increases due to constrained resources and limited treatment options [2].

Again, our analysis indicates that the amount of time spent travelling affects total costs associated with AMR infections. Generally, the longer it takes to get medical care, the higher the costs incurred by the patient and/or guardian. Safdar et al. conducted a study in Pakistan to find the impact of economic burden of AMR on patients and found that direct costs like transport among other costs, added an additional burden of US$12.30 for AMR cases compared to non-infected controls [44]. A related study on exploring the economic impact of inappropriate antibiotic use in Ghana found an increase in total costs due to direct travel costs and lost income due to travel which together were estimated to be around US$44 million [45]. All these reported findings from previous studies agree with this study findings. This indicates that geographic access to healthcare is a crucial determinant of economic outcomes in the context of AMR. Improving transportation infrastructure and providing more localized healthcare services could reduce travel-related costs and improve access to timely treatment.

There is evidence from this study that patients with bacterial resistant infections incur relatively higher costs than those with susceptible infections. Previous studies have reported a higher cost burden of resistant compared to susceptible infections [36]. This is particularly true for BSIs relative to UTIs in this study reflecting the greater severity and management complexity of BSIs, with costs significantly increasing when the causative bacteria are *K. pneumoniae* or when multiple bacteria are identified. This result closely relates with previous findings by Chandroulis et al., 2024, that total care costs were significantly higher for primary compared to secondary BSI, driven by longer hospital stays and increased antibiotic costs [46]. Previous studies also reported that BSIs can easily develop into sepsis and have a high mortality rate, requiring adequate treatment hence more costs [47,48]. In 2008, Rice identified ABR pathogens that were both highly virulent and resistant while investigating use of Federal funding for the study of AMR in nosocomial pathogens which included *K. pneumoniae* [49].

This study provides economic cost estimates to fill the knowledge gap regarding the uncertainty associated with resistant infection costs especially from a patient perspective. It is the first to explore the total economic costs involved at three different healthcare facilities in Malawi without external influence on funding models of the facilities such as provision of extra reagents. Also, it highlights the use and impact of key economic variables such lost earnings, health insurance, employment status, and travel-related costs in modelling total costs related to AMR infections in humans. The cost analysis literature for Malawi so far usually focused on assessing the clinical cost impact of AMR, cost of AMR surveillance systems, and cost-effectiveness of antibiotics using a specific case setting like a single hospital, single type of infection (BSI or UTI only), or isolated pathogens. The study also shows that AMR burden can be contextually different with limited resource settings facing the greatest challenge. The study reports a longer length of stay especially for resistant infections compared to many studies on AMR. Again, the use of IV regression in this study contributes to addressing the methodological debate that economic costs and AMR infections along with other important variables, are among the complex and multiple components of total costs. We agree that applicability of unique analytical techniques need to be checked if appropriate for reliable results to guide policies.

Understanding the economic costs of AMR is crucial to support Malawi NAP, mobilize resources and key stakeholders, and to design sustainable strategies to fight AMR. Currently, resources in the fight against AMR are largely provided by governments and a few donors such as the Fleming fund in the case of Malawi. Our economic cost analysis supports this cause by providing baseline cost data to be used by stakeholders and future studies investigating the economic cost burden using some variables in this study. We hope that efforts to mobilize resources for AMR will escalate given the abnormal economic financial burden faced by patients. Generally, the study can serve as a starting point for other researchers, mostly in LMICs, to estimate economic costs related to AMR using data problem-solving analytical techniques if found, in a broader sense of the human aspect in the one health setting.

### Study Limitations and Strengths

This study has some limitations. First, the sample size and geographical coverage, intended to ensure representativeness of the Malawian context, is not sufficiently large, but is statistically good enough for estimation. This is because most health care facilities did not have the capacity to culture blood or urine samples on their own for reliable test results. Attempts to use three other facilities proved difficult as they recruited at most two study participants in two months which was costly to the study. Again, taking advantage of typical hospital settings to reflect true costs by not providing any resources like reagents even during stockouts which were common, slowed the study recruitment further and hence the relatively lower sample size.

Second, the study failed to collect all data on antibiotic usage for the purposes of tracking whether there were significant changes in classes of antibiotics, say from first to second line antibiotics or vice-versa in relation to total costs, length of hospital stays, type of infection, and resistance. Patients’ registers were missing in some cases and even the patients could not recall, describe or know the antibiotics they had taken during treatment. This created data gaps which made the data unusable for this purpose. Future studies should consider including this information. Despite this challenge, the costing of antibiotics which were accounted for during data collection was done.

Third, the study’s scope has not taken into account co-infections, comorbidity, or the re-emergence of infections as key separate variables, which may have different cost structures if included. However, attempts to ask cost details on these were made during follow ups. We recommend attempting their inclusion in future studies for full costing of AMR.

Furthermore, this study did not conduct a cost-effectiveness analysis of costs due to the lack of intervention versus control variables that lead to AMR costs. Future studies should consider evaluating the long-term savings from investments in infection prevention and control with deliberate inclusion of intervening and control variables on health outcomes’ cost whenever possible. 

Another limitation is that the use of cross-sectional data limits the long-term impact of independent economic variable(s) influence on the dependent variable. Use of time-series or panel data along with qualitative data should be considered to improve the reliability of results with long-term impact. Complementary qualitative data will help understand the coping strategies employed by families during loss of income as a result of resistant infections, for instance selling assets, removing children from school.

Notwithstanding these limitations, the strengths of this study include the use of strong methodology for data analysis, specifically IV regression, which remedied the endogeneity problem found in the data, along with descriptive statistics. Also, the study has included most relevant economic cost variables related to AMR such as lost earnings, AMR-associated deaths, insurance, and economic status of individuals. The study further collects data from multiple sites while avoiding contamination of results among health facilities costs and patients’ data. The other strength of this study is the comparison of costs before and during follow-ups of patients for full economic cost capture.

## 5. Conclusions

This study provides a detailed micro-costing analysis of the costs associated with antimicrobial-resistant infections in a low-resource setting. The findings highlight the substantial financial burden of AMR, including mortality; identify key socio-economic drivers of cost; and underscore the critical challenge of endogeneity in econometric evaluations of AMR. Based on the findings and discussion, we propose the following multi-tiered recommendations:

First, national health policy and AMR steering committees should aim to strengthen financial protection by developing and subsidizing context-appropriate health insurance or waiver schemes targeting low-income populations to mitigate catastrophic out-of-pocket expenditures. This should be a cornerstone of the Malawi National Action Plan on AMR.

Second, AMR stewardship programs should trickle down to community healthcare facilities’ educational-based programs that emphasize the importance of health literacy, focusing on the relationship between education, health insurance, and access to care, especially antibiotics use. This should consider strategies to manage simpler AMR cases at lower-level health centres to reduce travel burdens and associated costs for patients through training and equipping mid-level providers. Such efforts should be made to include women and economically disadvantaged groups of beneficiaries to bridge the knowledge gap.

In addition, governments should aim to invest in transportation services to healthcare facilities through healthcare interventions such as universal health coverage, especially in rural areas, to reduce travel time and costs. One way is to consider partnerships with local transport providers to facilitate easier access to medical care especially for patients suffering from antibiotic-resistant infections.

Lastly, NAP and all stakeholders should encourage further research into the economic implications of AMR to inform policy decisions with emphasis on longitudinal studies, which could provide deeper insights into the long-term impacts on health systems and economies.

## Figures and Tables

**Table 1:** Descriptive statistics.

Variable	Mean/Proportion	Std Err	[95% CI]
Age (years)	41.6	0.80	40.01–43.20
Sex (*male*)	45	0.02	0.41–0.49
Location *(urban*)	65	0.02	0.61–0.69
Education level (*None*)	16	0.15	0.13–0.19
(*primary*)	43	0.02	0.39–0.47
(*secondary*)	29	0.02	0.26–0.33
(*tertiary*)	12	0.01	0.10–0.15
Occupation (*not employed*)	36	0.02	0.32–0.40
(*farming*)	29	0.02	0.26–0.33
(*non-farm business*)	21	0.02	0.18–0.24
(*civil servant*)	14	0.01	0.11–0.17
Bank account (*with account*)	48	0.02	0.44–0.52
Health insurance (*covered*)	06	0.01	0.04–0.08
Infection type (*BSI*)	49	0.02	0.45–0.53
Length of hospital stay	20.5	0.86	18.76–22.15
Pathogen (*P. aeruginosa*)	22	0.02	0.19–0.26
(*E. coli*)	43	0.02	0.39–0.47
(*S. aureus*)	10	0.01	0.07–0.12
(*K. pneumoniae*)	14	0.01	0.11–0.17
(*other/multiple*)	11	0.01	0.09–0.14
Resistance (*resistant*)	45	0.02	0.41–0.49
Mode_transport (*private vehicle*)	18	0.02	0.15–0.21
(*public vehicle*)	30	0.02	0.27–0.34
(*motorbike*)	33	0.02	0.30–0.37
(*bicycle*)	06	0.01	0.04–0.08
(*walked*)	10	0.01	0.08–0.13
(*other*)	03	0.01	0.02–0.04
Travel time (*less than one hour*)	62	0.02	0.59–0.67
(*one to two hours*)	25	0.02	0.22–0.29
(*more than two hours*)	10	0.01	0.07–0.12
(*about five hours*)	02	0.01	0.01–0.03
(*more than five hours*)	01	0.00	0.00–0.02
Transport costs	14,211.43	878.21	12,486.75–15,936.12
Lost earnings	112,625.60	22,900.59	67,651.87–157,599.30
Treatment costs	37,165.29	4066.95	29,178.33–45,152.25
Additional costs	47,697.27	5077.32	37,726.09–57,668.45
Death associated costs	253,040	35,840.69	179,068.50–327,011.50
Total costs	283,815.10	28,382.22	228,076.20–339,554.00

**Table 2:** This table shows the prevalence of pathogens versus the type of infection.

		Causative Pathogen				
		*E. coli*	*K. pneumoniae*	*S. aureus*	*P. aeruginosa*	Other
Infection type	UTI	52.87%	47.62%	58.62%	50.36%	48.31%
	BSI	47.13%	52.38%	41.38%	49.64%	51.69%
		n = 610	Pearson chi2(4) = 3.38			*p* < 0.497

**Table 3:** This table shows the categorization of types of resistant infections and pathogens.

	Susceptible Infection	Resistant Infection
*P. aeruginosa*	79.56% (n = 109)	20.44% (n = 28)
*Other*	35.71% (n = 25)	64.29% (n = 70)
*E*. *coli*	53.26% (n = 139)	46.74% (n = 122)
*S*. *aureus*	46.55% (n = 27)	53.45% (n = 31)
*K. pneumoniae*	42.86% (n = 36)	57.14% (n = 48)
n = 610	Pearson chi2(4) = 50.93	*p* < 0.000

**Table 4:** This table shows detailed summary statistics for direct medical, direct non-medical, and productivity cost categories.

Cost Category	n	Mean	SD	Median	IQR	Min	Max
Direct medical	610	37,165.29	100,446.40	2300	23,000	0	786,000
Direct non-medical	610	61,908.71	130,365.30	25,000	64,000	0	2,010,000
Productivity losses	610	160,322.80	614,469.30	40,000	127,000	0	13,100,000

**Table 5:** This table shows the IV 2SLS regression results.

ln (Total Costs)	Coef.	Std. Err
Resistance	0.8225 ***	0.1888
Age	0.0002	0.0165
Age-Squared	0.0001	0.0002
Health Insurance	−0.7464 *	0.3324
**Education Level**		
*Primary*	−0.1759	0.2136
*Secondary*	−0.1379	0.2410
*Tertiary*	−0.2340	0.3347
Bank account	0.3279 **	0.1486
**Occupation**		
*Farming*	0.1145	0.1880
*Business*	0.6639 ***	0.2100
*Civil Servant*	−0.4487 *	0.2665
*Other*	0.3491	1.0108
**Travel-duration**		
*1 to 2 h*	0.7555 ***	0.1694
*>2 h*	1.3628 ***	0.2540
*About 5 h*	1.2910 **	0.5317
*>5 h*	1.4185	0.8711
Infection type	0.9087 ***	0.1446
**Pathogen**		
*P. aeruginosa*	−0.2412	0.1907
*S. aureus*	−0.2597	0.2525
*K. pneumoniae*	0.6965 ***	0.2186
*Other*	0.7476 ***	0.2339
Constant	9.7864 ***	0.3632
Obs (N) = 609 Wald chi2(21) = 220.9 Prob > chi2 < 0.0000 R-Sqd = 0.2913 Root MSE = 1.6987		

Note: ***, **, and * denote statistical significance at 1%, 5%, and 10%, respectively.

**Figure A1:**
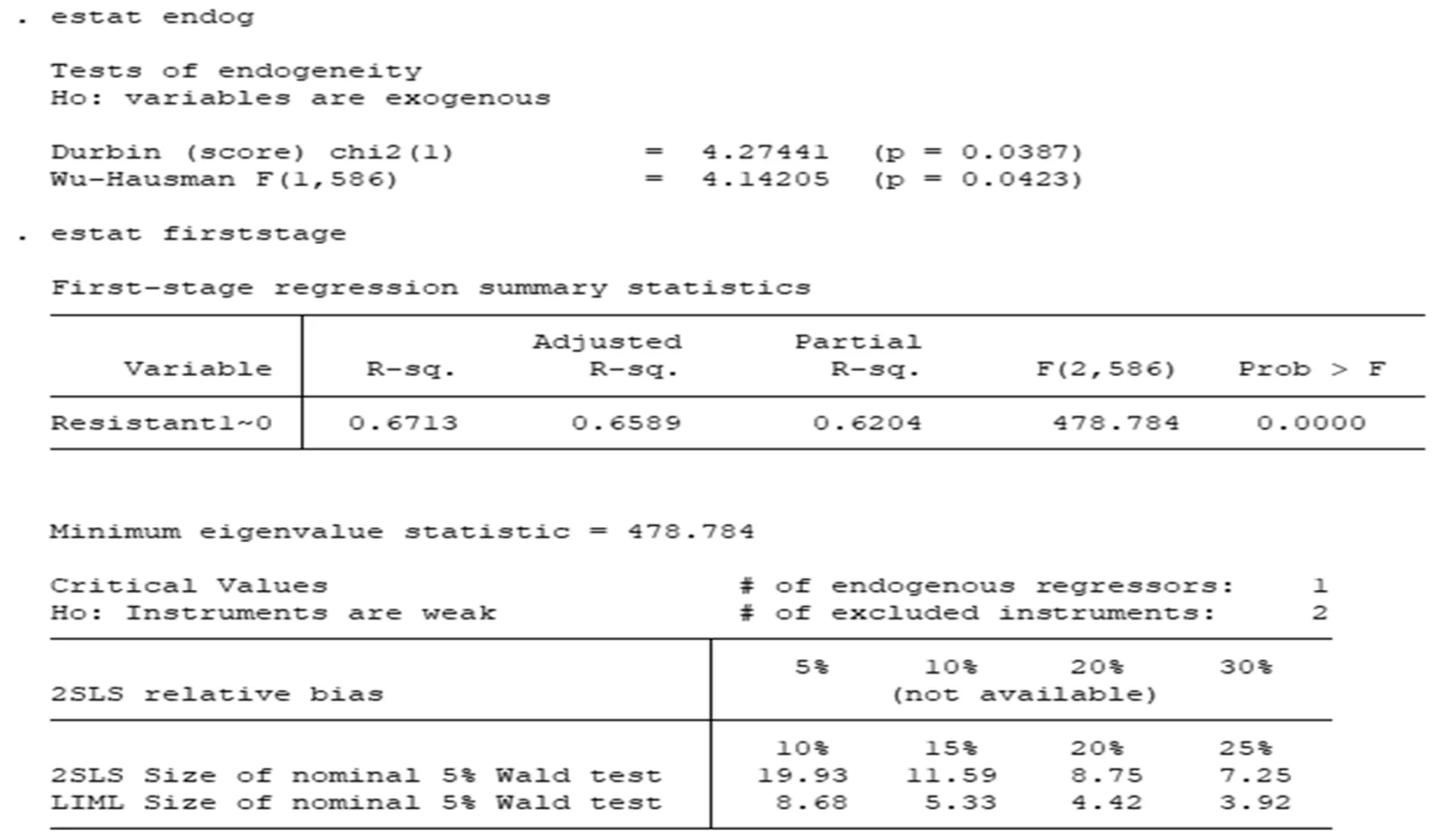
Durbin Chi-square and Wu-Husman tests.

**Figure A2:**
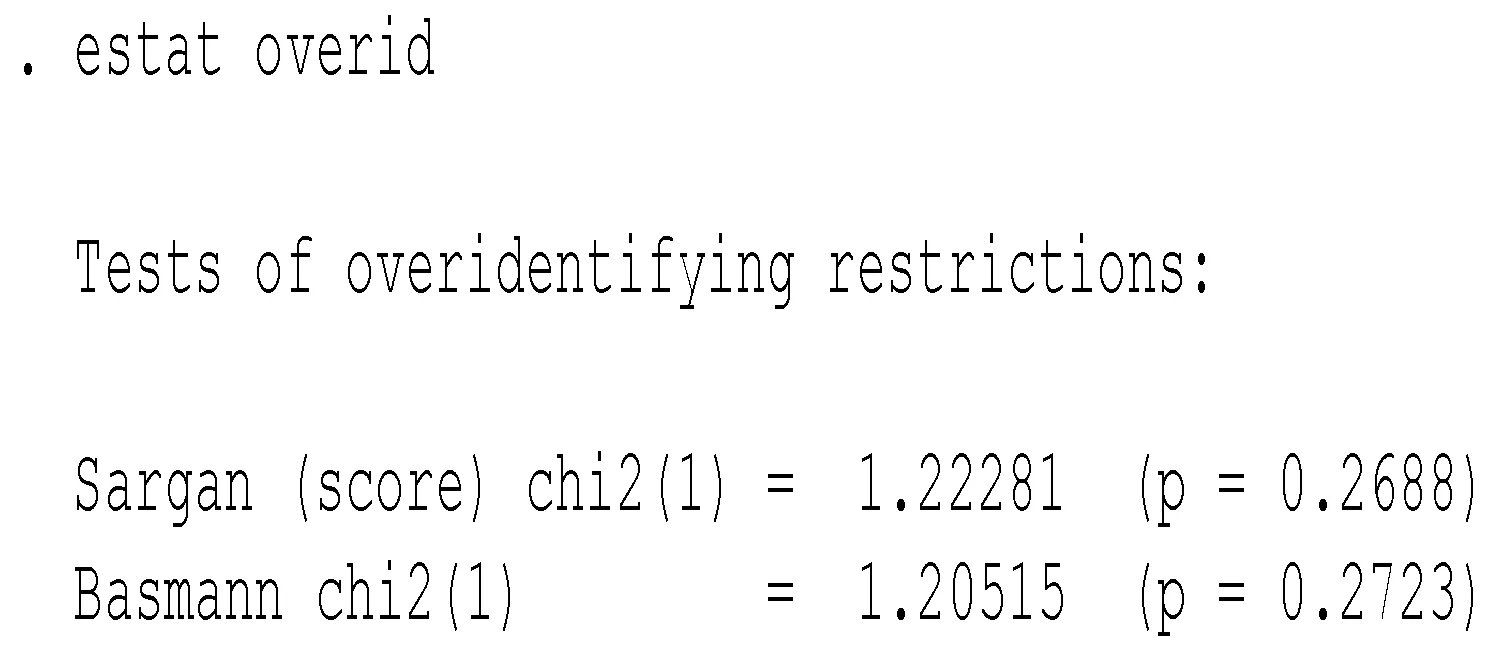
Sargan test.

## Data Availability

The data presented in this study are available from the corresponding authors upon reasonable request.

## Supplementary Materials

The following supporting information can be downloaded at: https://www.mdpi.com/article/10.3390/tropicalmed11100276/s1, Table S1: Stata IV 2sls regression.

## Abbreviations

AHHME	Agriculture Human Health Micro-Economic model
AMR	Antimicrobial resistance
APHRC	African Population Health Research Centre
ARB	Antimicrobial-resistant bacteria
BSI	Bloodstream infections
CDC	Centre for Disease Control
CI	Confidence interval
COMREC	College of Medicine Research Ethics Committee
ESBL	Extended Spectrum Beta-Lactamase
GDP	Gross Domestic Product
HICs	High-Income Countries
HIMS	Health information management system
ICUs	Intensive care units
IQR	Interquartile range
IV	Instrumental variable
JPIAMR	Joint Programming Initiative on Antimicrobial Resistance
LMICs	Low- and middle-income countries
MK	Malawi Kwacha
MRSA	Methicillin-resistant Staphylococcus aureus
NAP	National Action Plan
OECD	Organisation for Economic Co-operation and Development
QECH	Queen Elizabeth Central Hospital
SD	Standard deviation
SIDA	Swedish International Development Agency
USD	United States Dollars
UTI	Urinary tract infections
2SLS	2-stage least squares

## Appendix C

**Table A1:** EUCAST guidelines.

*ENTEROBACTERALES* NOTES; A. Ignore growth that may appear as a thin inner zone on some batches of MH				
ANTIBIOTIC	CONTENT	ZONE DIAMETER BREAKPOINTS		COMMENTS
		S≥	R<	
Ampicillin	10 μg	14	14	Note A
Amoxicillin-Clavulanic Acid (iv)	20–10 μg	19	19	
Amoxicillin-Clavulanic Acid (Oral)	20–10 μg	50	19	Other Indications (ATU 19–20)
Amoxicillin-Clavulanic Acid (UTI)	20–10 μg	16	16	Uncomplicated UTI Only
Piperacillin-tazobactam	30–6 μg	20	20	ATU = 19
Cefepime	30 μg	27	24	
Cefotaxime	5 μg	20	17	Indications other than meningitis
Cefotaxime (Meningitis)	5 μg	20	20	
Ceftriaxone	30 μg	27	24	Indications other than meningitis
Ceftriaxone (Meningitis)	30 μg	27	27	
Cefoxitin	30 μg	19	19	Screen Only
Ceftazidime	10 μg	22	19	
Cefixime (UTI)	5 μg	17	17	Uncomplicated UTI Only
Ertapenem	10 μg	23	23	
Meropenem	10 μg	22	16	Indications other than meningitis
Meropenem (Meningitis)	10 μg	22	22	
Gentamicin	10 μg	17	17	
Amikacin	30 μg	18	18	
Tobramycin	10 μg	16	16	Including uncomplicated UTI
Ciprofloxacin	5 μg	25	22	ATU = 22–24
Norfloxacin (UTI)	10 μg	24	24	Uncomplicated UTI Only
Trimethoprim-sulfamethoxazole Except Serratia	1.25/23.75 μg	15	15	
Nitrofurantoin (UTI)	100 μg	11	11	Uncomplicated UTI Only, *E. coli*
PSEUDOMONAS SPECIES				
Cefepime	30 μg	50	21	ATU= 19–23
Ceftazidime	10 μg	50	17	
Piperacillin-tazobactam	30–6 μg	50	18	ATU = 18–19
Meropenem	10 μg	20	14	Indications other than meningitis (*P. aeruginosa*)
Meropenem	10 μg	24	18	Indications other than meningitis (Pseudomonas other than *P. aeruginosa*)
Meropenem	10 μg	20	20	Meningitis (*P. aeruginosa*)
Ciprofloxacin	5 μg	50	26	
Amikacin	30 μg	15	15	
Tobramycin	10 μg	18	18	
ACINETOBACTER SPECIES				
Meropenem	10 μg	21	15	
Amikacin	30 μg	19	19	
Ciprofloxacin	5 μg	50	21	
Gentamicin	10 μg	17	17	
Tobramycin	10 μg	17	17	
Trimethoprim-sulfamethoxazole	1.25/23.75 μg	16	16	
STAPHYLOCOCCUS SPECIES				
Benzylpenicillin	1 unit	26	26	Isolates susceptible to benzylpenicillin and Cefoxitin are also susceptible to all penicillins.
Oxacillin	1 μg	20	20	Screen only
Cefoxitin (Screen only)	30 μg	22	22	Susceptivity of Staphylococci to Cephalosporins is inferred from the cefoxitin susceptibility except for Cefixime and Ceftazidime. Susceptibility of Staphylococci to Carbapenems is inferred from the cefoxitin susceptibility.
Ciprofloxacin	5 μg	50	17	*S. aureus*
Ciprofloxacin	5 μg	50	22	Coagulase-negative staphylococcus
Amikacin	30 μg	15	15	
Gentamicin (*S. aureus*)	10 μg	18	18	
Gentamicin	10 μg	22	22	Coagulase-negative staphylococcus
Tobramycin (*S. aureus*)	10 μg	18	18	
Tobramycin	10 μg	20	20	Coagulase-negative staphylococcus
Erythromycin	15 μg	21	21	Isolates susceptible to Erythromycin can be reported as susceptible to Azithromycin, Clarithromycin and roxithromycin.
Clindamycin	2 μg	22	22	
Tetracycline	30 μg	22	22	Isolates susceptible to Tetracycline can be reported as susceptible to Doxycycline and Minocycline.
Nitrofurantoin (UTI)	100 μg	13	13	Uncomplicated UTI Only, *S. saprophyticus*
Trimethoprim-sulfamethoxazole	1.25/23.75 μg	24	24	
STREPTOCOCCUS GROUPS A, B, C AND G NOTE: This group of bacteria includes many species, which can be grouped as follows; Group A: *S. pyogenes* Group C: *S. dysgalactiae* (plus more rarely isolated *S. equi*) Group B: *S. agalactiae* Group G: *S. dysgalactiae* and *S. canis* *S. dysgalactiae* includes the subspecies *equisimilis* and *dysgalactiae*, *S. equi* includes the subspecies *equi* and *zooepidemicus.*				
Benzylpenicillin	1 unit	23	23	Streptococcus groups A, C and G
Benzylpenicillin (Meningitis)	1 unit	18	18	*S. agalactiae (GROUP B Streptococci)*
Vancomycin	5 μg	13	13	
Erythromycin	15 μg	21	21	Isolates susceptible to Erythromycin can be reported as susceptible to Azithromycin, Clarithromycin and roxithromycin.
Clindamycin	2 μg	17	17	
Tetracycline	30 μg	23	23	Isolates susceptible to Tetracycline can be reported as susceptible to Doxycycline and Minocycline.
Nitrofurantoin (UTI)	100 μg	15	15	Uncomplicated UTI Only, *S. agalactiae*
Trimethoprim-sulfamethoxazole	1.25/23.75 μg	16	16	
STREPTOCOCCUS PNEUMONIAE NOTES: The oxacillin 1 μg disk diffusion screening test shall be used to exclude beta lactam resistance mechanisms. When the screen is Negative (Oxacillin zone diameter ≥ 20 mm), all beta lactam agents for which clinical breakpoints are available, including those with “Note” can be reported susceptible without further testing, except for Cefaclor, which if reported should be reported as “susceptible increased exposure” (I). When the screen is Positive (Oxacillin zone diameter < 20 mm), refer to flowchart in EUCAST.				
Oxacillin (Screen only)	1 μg	20	20	Note above
Vancomycin	5 μg	16	16	
Erythromycin	15 μg	22	22	
Clindamycin	2 μg	16	16	
Tetracycline	30 μg	25	25	
Trimethoprim-sulfamethoxazole	1.25/23.75 μg	15	15	
VIRIDANS GROUP STREPTOCOCCI NOTE: Place clindamycin and erythromycin discs 12–16 mm apart (edge to edge) and look for antagonism (the D phenomenon) to detect inducible clindamycin resistance.				
Benzylpenicillin	1 unit	21	12	
Cefepime	30 μg	25	25	
Cefotaxime	5 μg	23	23	
Ceftriaxone	30 μg	27	27	
Vancomycin	5 μg	15	15	
Clindamycin	2 μg	19	19	Note above
ENTEROCOCCUS SPECIES NOTE C: Susceptibility to Ciprofloxacin and levofloxacin can be inferred from the Norfloxacin disc diffusion screening				
Ciprofloxacin (UTI)	5 μg	15	15	Uncomplicated UTI Only
Piperacillin-tazobactam	30–6 μg	50	18	*E. faecalis*
Norfloxacin (Screen only)	10 μg	12	12	*NOTE C*
Vancomycin	5 μg	12	12	*E. faecalis* and *E. faecium*
Nitrofurantoin (UTI)	100 μg	15	15	Uncomplicated UTI Only, *E. faecalis*
HAEMOPHILLUS INFLUENZAE				
Benzylpenicillin (Screen Only)	1 unit	12	12	
Cefepime	30 μg	28	28	
Cefixime	5 μg	26	26	
Cefotaxime	5 μg	27	27	
Ceftriaxone	30 μg	32	32	
Ertapenem	10 μg	23	23	
Meropenem	10 μg	20	20	
Ciprofloxacin	5 μg	32	32	
Nalidixic Acid (Screen Only)	30 μg	23	23	For screening fluoroquinolone resistances
Tetracycline	30 μg	25	25	
Chloramphenicol	30 μg	28	28	
Trimethoprim-sulfamethoxazole	1.25/23.75 μg	23	23	
LISTERIA MONOCYTOGENES				
Benzylpenicillin	1 unit	13	13	
Meropenem	10 μg	26	26	
Erythromycin	15 μg	25	25	
Trimethoprim-sulfamethoxazole	1.25/23.75 μg	29	29	
CORYNEBACTERIUM SPECIES				
Benzylpenicillin	1 unit	50	12	
Ciprofloxacin	5 μg	50	25	
Vancomycin	5 μg	17	17	
Clindamycin	2 μg	20	20	
Tetracycline	30 μg	24	24	
CORYNEBACTERIUM DIPHTHERIAE AND CORYNEBACTERIUM ULCERANS				
Benzylpenicillin	1 unit	50	12	
Cefotaxime	5 μg	50	15	
Meropenem	10 μg	24	24	
Ciprofloxacin	5 μg	50	24	
Erythromycin	15 μg	24	24	
Clindamycin	2 μg	15	15	
Tetracycline	30 μg	24	24	
Trimethoprim-sulfamethoxazole	1.25/23.75 μg	23	23	
BACILLUS SPECIES				
Meropenem	10 μg	25	25	
Ciprofloxacin	5 μg	50	23	
Vancomycin	5 μg	10	10	
Erythromycin	15 μg	24	24	
Clindamycin	2 μg	17	17	
AEROMONAS SPECIES				
Cefepime	30 μg	27	24	
Ceftazidime	10 μg	24	21	
Ciprofloxacin	5 μg	27	24	
Trimethoprim-sulfamethoxazole	1.25/23.75 μg	19	16	
VIBRIO SPECIES NOTES:				
Susceptibility to azithromycin (and erythromycin when azithromycin is not available) is inferred from the erythromycin disk diffusion test.				
Susceptibility to doxycycline (and tetracycline when doxycycline is not available) is inferred from the tetracycline disk diffusion test.				
Piperacillin-tazobactam	30–6 μg	26	26	
Cefotaxime	5 μg	21	21	
Ceftazidime	10 μg	22	22	
Meropenem	10 μg	24	24	
Ciprofloxacin	5 μg	23	23	
Azithromycin	15 μg	16	16	
Erythromycin (Screen only)	15 μg	12	12	
Tetracycline (Screen only)	30 μg	20	20	
Trimethoprim-sulfamethoxazole	1.25/23.75 μg	21	21	
