# Supplementary material for: Estimating Economic Costs Associated with Treating Antimicrobial-Resistant Infections in Humans in Malawi
Source: Trop Med Infect Dis. 2026 Sep 30;11(10):276. doi: 10.3390/tropicalmed11100276 (PMC13709817; doi:10.3390/tropicalmed11100276)
Supplement: Supplementary file 1 [file tropicalmed-11-00276-s001.zip › tropicalmed-4511222-supplementary.pdf]

Table S1. Stata IV 2sls regression.

```
Instrumental variables (2SLS) regression          Number of obs   =        609
                                                  Wald chi2(21)   =     220.90
                                                  Prob > chi2     =     0.0000
                                                  R-squared       =     0.2913
                                                  Root MSE        =     1.6987
```

ln_costs	Coef.	Std. Err.	z	P>|z|	[95% Conf.	Interval]
Resistant1_Susceptible0	.8225167	.1887882	4.36	0.000	.4524985	1.192535
AgeYears	.0001788	.0165351	0.01	0.991	-.0322293	.0325869
Age_sqd	.0000653	.000181	0.36	0.718	-.0002895	.0004201
HealthInsuranceBeneficiaryYe	-.5925234	.3324325	-1.78	0.075	-1.244079	.0590323
HighestLevelofEducation						
Primary	-.1758734	.213635	-0.82	0.410	-.5945903	.2428435
Secondaary	-.1378707	.2410233	-0.57	0.567	-.6102677	.3345264
Tertiary	-.2340465	.3346738	-0.70	0.484	-.8899952	.4219022
Occupation						
Farming	.1145298	.1879662	0.61	0.542	-.2538771	.4829368
Non-farm Business	.6639354	.2100088	3.16	0.002	.2523257	1.075545
Civil Servant	-.4486959	.2665269	-1.68	0.092	-.971079	.0736873
Others	.3490948	1.010828	0.35	0.730	-1.632092	2.330282
Travel_Duration						
1 to 2 hours	.7554528	.1694225	4.46	0.000	.4233908	1.087515
> 2 hours	1.362777	.253965	5.37	0.000	.8650146	1.860539
about 5 hours	1.290994	.531664	2.43	0.015	.2489521	2.333037
> 5 hours	1.418472	.871129	1.63	0.103	-.2889091	3.125854
Has_Bank_acc	.3279269	.1485804	2.21	0.027	.0367146	.6191392
1.Infection_Type	.9087365	.1446355	6.28	0.000	.625256	1.192217
Cause						
none	-.2411532	.1907027	-1.26	0.206	-.6149235	.1326172
S.aureus	-.2596912	.2525283	-1.03	0.304	-.7546375	.2352552
K.pneumoniae	.6965169	.218577	3.19	0.001	.2681139	1.12492
Other (multiple)	.7475778	.233944	3.20	0.001	.2890559	1.2061
_cons	9.786373	.3632409	26.94	0.000	9.074434	10.49831

```
Instrumented:  Resistant1_Susceptible0
Instruments:   AgeYears Age_sqd HealthInsuranceBeneficiaryYe 1.HighestLevelofEducation
               2.HighestLevelofEducation 3.HighestLevelofEducation 1.Occupation 2.Occupation
               3.Occupation 4.Occupation 2.Travel_Duration 3.Travel_Duration
               4.Travel_Duration 5.Travel_Duration Has_Bank_acc 1.Infection_Type 0.Cause
               2.Cause 3.Cause 4.Cause Illness_Duration123456 resisIlduration
```
